# Causes, Prevention, and Correction of Complications of Primary and Revision Septorhinoplasty

**DOI:** 10.7759/cureus.20557

**Published:** 2021-12-21

**Authors:** Mohammed Elsayed, Abdullah S Alghamdi, Mohammed Khan, Ammar Habibullah, Mohammad A Alshareef, Hosam Senan, Safiyah Hazazi, Ayan A Alqurashi, Futun G Alosiami

**Affiliations:** 1 Otolaryngology - Head and Neck Surgery, King Abdullah Medical City, Makkah, SAU; 2 Otolaryngology - Head and Neck Surgery, Al-Hada Armed Forces Hospital, Al-Taif, SAU; 3 Otolaryngology - Head and Neck Surgery, King Abdulaziz Medical City, Jeddah, SAU; 4 Otolaryngology - Head and Neck Surgery, Al-Noor Specialist Hospital, Makkah, SAU; 5 Otolaryngology, Umm Al-Qura University, Makkah, SAU

**Keywords:** revision rhinoplasty, facioplasty, king abdullah city hospital, complications, septorhinoplasty, experience

## Abstract

Background: Rhinoplasty is one of the most challenging esthetic operations as it demands an optimal esthetic and practical outcome. Complications of rhinoplasty may occur intraoperatively or postoperatively during wound healing and contracture.

Objectives: The aim of this study was to assess the complications of septorhinoplasty at King Abdullah Medical City Hospital (KAMCH) and to evaluate the satisfaction scores of the patients and the doctors after primary and revision septorhinoplasty.

Materials and methods: In the last five years, 32 out of 425 patients (7.5%) underwent revision septorhinoplasty to correct complications of the previous operations performed at KAMCH.

This is a retrospective single descriptive study that included Saudi patients aged 18 years and above who underwent primary and revision septorhinoplasty at KAMCH from January 2015 to March 2020. We reviewed the medical records of the patients to identify postoperative complications. Data were analyzed using SPSS statistical program (versions 7 and 8; SPSS Inc, Chicago).

Results: The mean age of the 32 patients who underwent revision septorhinoplasty was 26 ± 8.5 years. Most of the complications involved the nasofrontal angle and the columellolabial angle. Statistically significant improvements in the satisfaction scores of the patients and the doctors were observed before the first surgery, after the first surgery, and after the second surgery (P = 0.000 for each time point).

Conclusion: The satisfaction levels of the patients and the doctors improve after the second surgery.

## Introduction

The nose is the most noticeable facial feature, and its contribution to facial appearance is greater than that of any other facial feature. Besides its esthetic features, the nose is an essential organ of breathing and facial expression. In addition to function and esthetics, there is a psychological aspect to the nose in terms of the reaction of a patient and other people to the nose of the patient [1]. Hence, great attention is necessary when performing transformative surgery on such an important organ. Rhinoplasty is a surgical procedure with dual function: nose reconstruction and maintenance or improvement of the airway function of the nose [2]. Recently, the rate of rhinoplasty procedures in Saudi Arabia has increased; according to reports, rhinoplasty represents 30% of all esthetic procedures in Saudi Arabia [3]. The patients require rhinoplasty for acquired or congenital problems and for functional or esthetic reasons or both [4]. These problems may involve any part of the nasal anatomy [5]. The nose is classified into the following separate parts: the bony dorsum, cartilaginous dorsum, soft tissue, and skin [6].

Rhinoplasty is one of the most challenging, complicated, and unpredictable cosmetic operations [7]. Consequently, many complications may arise from rhinoplasty. General complications include bleeding, scarring, infection, septal perforation, prolonged edema, deformity, and nasal airway obstruction and the need for revision surgery [8]. Further, the most common acute complications of rhinoplasty are pain, edema, and periorbital ecchymosis [8]. Although some of the complications are temporary postoperative complications of septorhinoplasty, the fear of the patients for these complications is greater than estimated because the nose is the most noticeable facial feature [9].

The Rhinoplasty Outcome Evaluation (ROE) questionnaire developed by Alsarraf et al. is one of the most used tools for estimating patient satisfaction and surgical outcomes. This validated survey consists of six questions assessing social, emotional, and psychological variables. However, the ROE questionnaire focuses more on esthetics [10].

There is a lack of studies from Saudi Arabia on the complications of septorhinoplasty. The aim of this retrospective study was to determine the frequency of common and rare complications of septorhinoplasty and the methods that can be used to prevent and treat the complications.

## Materials and methods

Septorhinoplasty is considered one of the most common and most difficult facial cosmetic operations, and complications may occur in most cases. Based on their etiology, prevention, and treatment, the complications can be classified as common or rare.

In nearly every case, rhinoplasty is performed to improve and conserve the balance and appearance of the nose and face to achieve satisfactory results for the patient and the surgeon, while simultaneously preserving and optimizing the important functions of the nose by improving breathing and reducing airway obstruction or snoring. The basic principles of this surgery include careful analysis of the surgical problem, careful planning of procedures based on accurate anatomical knowledge, precision of surgical techniques, and minimal traumatic handling of tissues. Alteration, coverage, and transfer of skin and associated tissues are the most common procedures performed during rhinoplasty. Nevertheless, complications occur in most cases; thus, it is necessary to have sound knowledge of these complications to adequately prevent them.

This is a retrospective single descriptive study conducted during the period from January 2021 to April 2021 that included Saudi patients aged 18 years and above who underwent primary and revision septorhinoplasty at King Abdullah Medical City Hospital (KAMCH) from January 2015 to March 2020. Patients less than 18 years of age and patients who underwent rhinoplasty only for esthetic purposes were excluded from the study. We reviewed the medical records of all the patients, determined if they underwent septorhinoplasty, and documented postoperative complications to ascertain the rate of each complication.

In the last five years, 32 out of 425 patients (7.5%) underwent revision septorhinoplasty to correct complications of the previous operations performed at KAMCH.

We measured the rates of satisfaction of the patients and the doctors with esthetics, breathing, and olfaction. The data collection forms used did not contain nominative information. The patients were identified using serial study codes and initials, which were linked to patient names and medical record numbers in a separate identification log sheet and kept safely under lock and key. Data entry was performed by the research team. After verification, data were transferred directly to the statistical database

Common complications of septorhinoplasty

Nasal asymmetry and side-wall concavity (43% and 37% of cases, respectively) can be divided according to external anatomical parts of nose into bony vault (side-wall bony concavity), middle vault (side-wall cartilaginous concavity), and the tip . Asymmetry of the bony vault is a result of asymmetrical osteotomies and can be prevented by meticulousness during osteotomies. If it happened, it can be corrected by external percutaneous osteotomy using unilateral spreader graft at the depressed side and free diced or morselized conchal cartilage graft at the concave bony side. Asymmetry of the middle vault is usually caused by unmasked dorsal septal deviation after dorsal reduction and can be prevented by recognition of septal deviation. Correction of this type of complications can be done by use of perforated perpendicular plate of the ethmoid to correct the concave side of the septum or the use of crushed cartilage graft to camouflage the side-wall cartilaginous concavity or free diced cartilage injection. Asymmetry of the tip is caused due to asymmetric tip sutures and unmasked caudal septal deviation and it can be prevented by meticulous suture technique and inspection. Treatment is revision, placement of septal extension graft, or repositioning of caudal septum with swinging door and securing to nasal spine with suture.

Alar retraction (40% of cases) is defined as a cephalic elevation of the alar margin, which may result in excess nostril show. Correction by placement of alar rim grafts can restore support and create a more triangular nasal base. These grafts are long narrow cartilaginous grafts that are placed into precise pockets along the alar rim just caudal to the marginal incision. They measure 2 mm to 3 mm in thickness and width and 5 mm to 8 mm in length. Softer material, such as cartilage harvested from the ear or from the cephalic trim of the lower lateral cartilages, is preferable. The medial aspect of these grafts can be gently bruised to aid with camouflage. They can be secured to the surrounding soft tissue or to the lateral aspect of a shield graft using a 6.0 polydioxanone suture.

Dorsal irregularity (34% of cases) arises after contour irregularities are created along the bony and/or cartilaginous dorsum due to improper spreader graft or from irregularity after rasping if a bone spicule is not washed off with saline during operation. Usually dissection under the superficial musculoaponeurotic system (SMAS) layer and under the mucoperichondrial/mucoperiosteal flap is mandatorily performed during primary and revision septorhinoplasty after rasping and washing with saline to avoid asymmetry during bilateral spreader graft placement. Correction of dorsal irregularity requires smoothening or camouflaging any irregularities and restabilizing the middle vault structure. If the skin is too thin, temporalis fascia can be used as a blanket to camouflage any irregularity.

Alopecia (31% of cases) is due to the harvesting of the temporalis fascia for correction of dorsal nasal irregularity to make the skin thicker and to avoid the appearance of irregularity, especially in men. It is prevented by avoiding temporalis fascia harvesting in men, and dissect the skin of the nose under the SMAS layer. If it happens, transfer the male patient to the dermatology team for alopecia treatment.

A persistently wide tip after primary rhinoplasty (21% of cases) may be due to failure of the surgeon to account for a thick inelastic skin-soft tissue envelope when modifying the dome region. A common error of omission leading to a persistently wide tip is the failure to straighten convex lateral crura. Dome narrowing will not result in a defined triangular tip appearance if the lateral walls are curving outward. Unless the curvature is straightened with a suture technique or lateral crural struts, there will be a persistently wide tip. This complication can be detected from the basal view of the nose. When using lateral crural struts, strong segments of cartilage are required to overcome the curvature of the existing alar cartilage. After separating the vestibular skin from the undersurface of the lateral crura, the grafts are sutured to the undersurface of the curved cartilages. The caudal attachment of the lateral crus and skin should remain intact to prevent caudal migration of the graft.

Saddle nose deformity (15% of cases) is a multi-etiological condition associated with destabilization or destruction of the bony or cartilaginous structures of the nose. It is caused mainly due to loss of anterior septal cartilage between the rhinion (keystone area) and the "septal pedestal" at the level of the premaxilla and anterior nasal spine and it prevented with meticulous work at the keystone area. Saddle nose deformity can be difficult to correct and is best prevented. Stable reconstruction of the cartilaginous septum is the critical challenge in the operative treatment of saddle nose deformity. Mild saddle nose deformity may be corrected with crushed cartilage camouflage dorsal onlay grafting. If disarticulation of the keystone is noted intraoperatively, rib cartilage graft may be used for reconstruction.

The internal nasal valve is an anatomical region bounded laterally by the caudal margin of the upper lateral cartilage, medially by the nasal septum and floor of the nose, and inferiorly by the head of the inferior turbinate [6]. Internal nasal valve dysfunction (9% of cases) is a common secondary complication of rhinoplasty. It is caused by severely deviated strut, and surgery on the scroll ligament with over-resection of the lateral cartilages results in weakness of the internal nasal valve area during hump removal [7]. It is usually diagnosed when excessive medialization of the caudal margin of the upper lateral cartilage is observed with negative pressure during nasal inspiration [6]. For prevention, the adhesion is cut and collapse of the internal nasal valve is carefully corrected with the aim of repositioning the upper lateral cartilages. Correcting the collapse of the internal nasal valve is usually aimed at repositioning the upper lateral cartilages or placing a spreader graft, regardless of the type (auto, mini, or classic) or source (septum, concha, or rib) [6]. The significant technical detail is to position the graft such that the collapsed triangular cartilages can be raised and repositioned and the function of the internal nasal valve can be restored, thereby increasing its cross-sectional area [7].

Pneumothorax (9% of cases) develops when air enters the pleural space. There are two classifications of pneumothorax, namely primary spontaneous and secondary. Secondary pneumothorax is further classified into non-iatrogenic traumatic pneumothorax, usually managed with chest tube placement, and iatrogenic pneumothorax, which occurs during harvesting of the seventh rib in men and the fifth rib in women due to pleural tear during rib harvesting for nose reconstruction. Out of 20 patients with severe saddle nose deformity, pneumothorax during rib harvesting occurred in three patients (two men and one woman). For prevention of this during rib harvesting, sharp instruments are avoided to preserve the mucoperichondrium. Postoperative chest X-ray is mandatory, and a chest tube is placed until no air is left in the pleura.

Infections (four out of 32 cases) following septorhinoplasty are rare because the nose is well vascularized. Insufficient blood supply may be due to poor surgical technique, excessive cauterization, or systemic diseases such as diabetes. In our study, one year after surgery, four out of 32 patients were found to have infection due to permanent suture with 6.0 Prolene at the tip. We removed the suture and secured the tip with absorbable 4.0 Vicryl. In three other patients, human fascia lata was used to repair septal perforation and augment saddle nose deformity. During reoperation, the tutoplast was removed and conchal cartilage was reinserted to manage previous saddle nose deformity. The most common organisms that cause infection are gram-positive and gram-negative *Staphylococci*, *Streptococci*, *Hemophilus influenza*, and *Klebsiella pneumoniae*. A broad-spectrum penicillin and cephalosporin can be administered to prevent infection.

Statistical analysis

Data were analyzed using SPSS statistical program (versions 7 and 8; SPSS Inc, Chicago). Data summarization was presented using tables and graphs. Categorical variables were expressed as percentages. Numerical data were expressed as means and standard deviations (SDs) or as medians and ranges, depending on the type of distribution of the variable. The satisfaction scores of the patients and the doctors ranged from 1 to 5 (1 = very bad, 2 = bad, 3 = no change, 4 = good, 5 = excellent).

Ethical consideration

Ethical approval for our research was granted by the ethics review committee of KAMCH. The privacy of the collected data was maintained at all times. All data were saved in a safe place and are available only to the researchers. Each patient signed an informed consent form before study commencement.

## Results

Table 1 shows the satisfaction scores of the doctors regarding septorhinoplasty. There was a total of 33 participants in this study. Their mean age was 26 ± 8.5 years, and their median age was 23 years. The mean satisfaction score before the first surgery was 2.91 ± 0.63, and the median satisfaction score was 3, which indicates no changes. The mean satisfaction scores after the second and third surgeries were 3.58 ± 1.2 and 3.55 ± 0.9, respectively. The median satisfaction score after the second and third surgeries was 4, which indicates bad satisfaction.

**Table 1:** Doctor satisfaction

Variables	Participants (n = 33)			SD	Variance	Min.	Max.	Percentile		
	Mean	Median	Mode					25	50	75
Age (years)	26	23	18	8.5	72.48	16	46	18.75	23	31
Before first surgery	2.91	3	3	0.63	0.398	1	4	3	3	3
After first surgery	3.58	4	4	1.2	1.43	1	5	3	4	4
After second surgery	3.55	4	4	0.90	0.818	1	4	3.5	4	4

Table 2 shows the satisfaction scores of the patients regarding septorhinoplasty. A total of 32 patients participated in our study, and 10 patients were excluded because we could not contact them. The mean patient age was 25.27 ± 8.23 years, and the median age was 23 years. The mean patient satisfaction scores for esthetics, breathing, and olfaction before the first surgery were 2.05 ± 1.21, 3 ± 1.60, and 3.55 ± 1.40, respectively, and the corresponding median scores were 1 (very bad), 3 (no change), and 4 (good), respectively. Further, the mean patient satisfaction scores for esthetics, breathing, and olfaction after the first surgery were 3.18 ± 1.09, 3.91 ± 1.26, and 4.32 ± 1.08, respectively, and the corresponding median scores were 3 (no change), 4 (good), and 5 (excellent), respectively. The mean patient satisfaction scores for esthetics, breathing, and olfaction after the second surgery were 3.41 ± 1.22, 4.23 ± 1.15, and 4.50 ± 0.859, respectively, and the corresponding median scores were 3 (no change), 4 (good), and 5 (excellent), respectively.

**Table 2:** Patient satisfaction

Variables	Participants (n = 22)					SD	Variance	Min.	Max.	Percentile		
	Mean	Median		Mode						25	50	75
Age (years)	25.27	23		18		8.23	67.7	16	46	18	23	30
Before first surgery												
Esthetics	2.05		2		1	1.21	1.47	1	5	1	2	3
Breathing	3		3		5	1.60	2.57	1	5	1.75	3	5
Olfaction	3.55		4		5	1.40	1.97	1	5	2	4	5
After first surgery												
Esthetics	3.18		3		3	1.09	1.20	1	5	2	3	4
Breathing	3.91		4		5	1.26	1.61	1	2	3	4	5
Olfaction	4.32		5		5	1.08	1.18	1	5	3.75	5	5
After second surgery												
Esthetics	3.41		3		3	1.22	1.49	1	5	3	3	4.25
Breathing	4.23		5		5	1.15	1.32	1	5	3.75	5	5
Olfaction	4.50		5		5	0.859	0.738	2	3	4	5	5

Table 3 shows patient characteristics. Data for all 32 participants were available. We evaluated the radix-tip distance, basal breadth, dorsal breadth, interpapillary distance, nasofrontal angle, and columellolabial angle before and after the first surgery and after the second surgery. The nasofrontal angle and columellolabial angle had the highest values at the three timepoints. The mean nasofrontal angles before the first surgery, after the first surgery, and after the second surgery were 137.8 ± 12.01 degrees , 135.9 ± 12.7 degrees, and 135.6 ± 13.83 degrees, respectively. Further, the mean columellolabial angles before the first surgery, after the first surgery, and after the second surgery were 92.07 ± 15.03 degrees, 92.3 ± 12.3 degrees, and 94.48 ± 12.5 degrees, respectively. A quick overview of our results revealed a reduction in most complications after the first surgery.

**Table 3:** Patient characteristics

Variables	Participants (n = 32)					SD	Variance	Min.	Max.	Percentile		
	Mean		Median	Mode						25	50	75
Before first surgery												
Radix-tip	1.98	1.97			1.80	0.237	0.057	1.61	2.43	1.80	1.97	2.14
Basal breadth	1.36	1.33			1.24	0.191	0.037	1	1.83	1.24	1.33	1.47
Dorsal breadth	0.744	0.744			0.790	0.193	0.037	0.480	1.60	0.617	0.74	0.81
Interpapillary distance	2.99	2.99			2.99	0.000	0.000	0 .000	2.99	2.99	2.99	2.99
Nasofrontal angle	137.8	140.6			147.7	12.01	144.40	105.6	157	128.6	140.6	147.6
Columellolabial angle	92.07	92.81			90	15.03	255.91	60	119.2	82.3	92.8	100.6
After first surgery												
Radix-tip	1.88	1.84			1.80	0.228	0.052	1.51	2.49	1.69	1.84	2.05
Basal breadth	1.27	1.23			1.19	0.156	0.025	1.01	1.64	1.16	1.23	1.38
Dorsal breadth	0.760	0.770			0.770	0.108	0.012	0.500	0.980	0.695	0.770	0.85
Interpapillary distance	2.99	2.99			2.99	0.000	0.000	0.000	2.99	2.99	2.99	2.99
Nasofrontal angle	135.9	137.5			143.0	12.7	163.4	104.4	159.8	128.5	137.5	143.8
Columellolabial angle	92.3	92.1			90	12.3	153.6	65.8	119	83.3	92.17	101.7
After second surgery												
Radix-tip	1.87	1.86			1.72	0.233	0.055	1.48	2.33	1.69	1.86	2.06
Basal breadth	1.30	1.31			1.30	0.165	0.027	1.03	1.67	1.16	1.31	1.43
Dorsal breadth	0.74	0.740			0.850	0.116	0.014	0.500	1.03	0.640	0.740	0.842
Interpapillary distance	2.99	2.99			2.99	0.000	0.000	0.000	2.99	2.99	2.99	2.99
Nasofrontal angle	135.6	140.9			146	13.83	191.2	103.2	156.9	124.0	140.9	145.5
Columellolabial angle	94.48	96.33			90	12.5	158.3	65	118	87.41	96.33	101.54

Table 4 shows the results of the statistical analysis that compared the satisfaction scores of the patients and the doctors. The results showed a statistically significant association between the satisfaction scores of the patients and the doctors before the first surgery, after the first surgery, and after the second surgery (P = 0.000 for each timepoint).

**Table 4:** Comparison of means CI: confidence interval

Scale	t	df	Significance (two-tailed)	Mean difference	95% CI of the difference	
					Lower	Upper
Before first surgery	78.93	31	0.000	39.50	38.48	40.52
After first surgery	73.04	31	0.000	39.20	38.10	40.29
After second surgery	67.04	31	0.000	39.51	38.31	40.71

## Discussion

Facial anatomy, especially as pertains the nose, is of great importance to beauty. Thus, nasal cosmetic surgeries (including rhinoplasty) can significantly affect a person's beauty and facial proportion. Rhinoplasty is the most common cosmetic operation performed by plastic surgeons. It is reported to have a weaker correlation with satisfaction level than other cosmetic operations [11]. The aim of this study was to determine the satisfaction levels of the patients and the doctors regarding primary and revision septorhinoplasties performed at KAMCH for the purpose of minimizing complications.

Revision rhinoplasty is more challenging than primary rhinoplasty because its principal goal is to eliminate functional or static defects after a failed prior surgery and to satisfy patient expectations [12]. Our results are consistent with those of earlier studies that reported higher patient and doctor satisfaction levels after the second surgery than before or after the first surgery. However, our results differ from those of the study by Saleh et al., who evaluated post-rhinoplasty satisfaction and quality of life. They reported that the patients have significantly high post-rhinoplasty satisfaction levels [13]. Rezaei et al. [14] also reported significantly high post-rhinoplasty satisfaction levels among the patients. Post-rhinoplasty satisfaction levels were also reported to be higher in the third month than in the first month. To optimize patient satisfaction after revision surgery, surgeons must be aware of the esthetic and functional complaints of the patients and must perform thorough nasal evaluations to ensure that all aspects of the nasal anatomy are excellent [15].

Our results show a reduction in most complications after the first surgery and further improvement after the second surgery in terms of basal breadth and columellolabial angle.

In this study, a statistically significant association was observed between the patient and the doctor satisfaction scores before the first surgery, after the first surgery, and after the second surgery. These results are not consistent with those of the study by Rezaei et al. [14], who reported no significant association between the patient and the doctor satisfaction scores.

In our study, due to limitations such as low number of referrals and similarity of cases, our sample size for comparison was not large. Therefore, it is advisable to use a larger sample size in future studies. Moreover, multiple evaluations of the satisfaction level over longer time intervals have been suggested.

## Conclusions

In this study, the satisfaction levels of the patients and the doctors were assessed before and after the first septorhinoplasty and after the second surgery. In all 32 cases of revision septorhinoplasty, the patient and the doctor satisfaction levels increased after the second surgery.

## References

[1] Sinha V, Pillai P, George A, Memon R, Arya A, Shah S (2006). Rhinoplasty - our experience. Indian J Otolaryngol Head Neck Surg.

[2] Ishii LE, Tollefson TT, Basura GJ (2017). Clinical practice guideline: improving nasal form and function after rhinoplasty. Otolaryngol Head Neck Surg.

[3] Alharethy SE (2017). Trends and demographic characteristics of Saudi cosmetic surgery patients. Saudi Med J.

[4] Bagheri SC, Khan HA, Jahangirnia A, Rad SS, Mortazavi H (2012). An analysis of 101 primary cosmetic rhinoplasties. J Oral Maxillofac Surg.

[5] Kienstra M (2011). Secondary rhinoplasty: revising the crooked nose. Facial Plast Surg.

[6] Foda HM, Magdy EA (2006). Combining rhinoplasty with septal perforation repair. Facial Plast Surg.

[7] Abbas OL (2016). Revision rhinoplasty: measurement of patient-reported outcomes and analysis of predictive factors. Springerplus.

[8] Sari E, Simsek G (2015). Comparison of the effects of total nasal block and central facial block on acute postoperative pain, edema, and ecchymosis after septorhinoplasty. Aesthetic Plast Surg.

[9] Youssef A, Ahmed S, Ibrahim AA, Daniel M, Abdelfattah HM, Morsi H (2018). Traumatic cerebrospinal fluid leakage following septorhinoplasty. Arch Plast Surg.

[10] Bulut OC, Wallner F, Oladokun D (2018). Long-term quality of life changes after primary septorhinoplasty. Qual Life Res.

[11] Torres S, Marianetti T (2016). Management of Common Complications in Rhinoplasty and Medical Rhinoplasty. A Textbook of Advanced Oral and Maxillofacial Surgery Volume 3.

[12] Boenisch M (2011). Complications in Rhinoplasty. Rhinoplasty Archive. https://www.rhinoplastyarchive.com/articles/rhinoplasty-fundamentals/complications-in-rhinoplasty.

[13] Sena Esteves S, Gonçalves Ferreira M, Carvalho Almeida J, Abrunhosa J, Almeida E Sousa C (2017). Evaluation of aesthetic and functional outcomes in rhinoplasty surgery: a prospective study. Braz J Otorhinolaryngol.

[14] Rezaei F, Rezaei F, Abbasi H, Moradi H (2019). A comparison of doctor/patient satisfaction with aesthetic outcomes of rhinoplasty: a prospective study. J Med Life.

[15] Saleh AM, Younes A, Friedman O (2012). Cosmetics and function: quality-of-life changes after rhinoplasty surgery. Laryngoscope.

